# Effect of soft tissue volume on midfacial gingival margin alterations following immediate implant placement in the esthetic zone: a 1-year randomized clinical and volumetric trial

**DOI:** 10.1186/s12903-024-04845-y

**Published:** 2024-10-19

**Authors:** Ahmed Ibrahim Aboul Fettouh, Noha Ayman Ghallab, Khaled Abdel Ghaffar, Azza Ezz Elarab, Nourhan Gamal Abdel-Aziz, Nael Adel Mina, Nesma Mohamed Shemais, Omnia Aboul Dahab

**Affiliations:** 1 Faculty of Dentistry, Misr International University, Cairo, Egypt; 2 Faculty of Dentistry, Cairo University, Cairo, Egypt; 3 Minister of Health, Egypt and Faculty of Dentistry, Ain Shams University, Cairo, Egypt; 4 Al-Ahram Canadian University, Cairo, Egypt and Faculty of Dentistry, Cairo University, Cairo, Egypt; 5 MSc. Faculty of Dentistry Cairo University, Cairo, Egypt; 6 BDS Misr International University, Cairo, Egypt

**Keywords:** Immediate implant, Connective tissue graft, Bone graft, Customized healing abutment, Esthetic zone

## Abstract

**Background:**

The current trial evaluated the midfacial gingival margin changes, volumetric, radiographic and clinical alterations 1-year following immediate implant placement with customized healing abutment (IIP + CHA) either solely, or in combination with xenograft (IIP + Bonegraft) or with connective tissue grafting (IIP + CTG) at sites with thin labial bone in the esthetic zone.

**Methods:**

Thirty-nine non-restorable maxillary teeth indicated for extraction in the esthetic zone were included. Participants were randomly assigned into three equal group; IIP + Bonegraft (Control), IIP + CTG and IIP + CHA. Midfacial gingival margin changes(mm) as primary outcome, labial soft tissue contour change(mm), interdental tissue height changes and total volume(mm^3^) were assessed. Amount of bone labial to the implant and crestal bone level changes were also recorded. All outcomes were measured 1-year post-operative.

**Results:**

The midfacial gingival margin changes demonstrated a significant difference (*P* ≤ 0.05) between the groups showing -0.98, -0.74 and -1.54 mm in sites treated with IIP + Bone graft, IIP + CTG and IIP + CHA respectively after1-year. While labial soft tissue contour change (mm), total volume (mm^3^) and distal interdental tissue height changes (mm) revealed a significant difference after one-year between the studied groups, yet mesial interdental tissue height changes showed no difference (*P* > 0.05). Both IIP + Bone graft and IIP + CHA groups revealed a significant positive correlation between the total volume loss (mm^3^) after 1 year and mid-facial gingival margin changes (*P* ≤ 0.05). However, no significant correlation was observed in the IIP + CTG group (*P* = 0.63). CBCT measurements showed a significant difference in crestal bone changes between the three groups (*P* ≤ 0.05), yet, there was no significant difference regarding mean amount of bone labial to the implant(*P* > 0.05).

**Conclusions:**

This investigation suggests that the mere presence of CTG simultaneous with IIP in the anterior maxilla reduced the midfacial gingival margin alterations (mm), besides, CTG decreased the overall volume loss (mm^3^) by 5-folds compared to the other studied groups after one year. Meanwhile, using CHA alone with IIP failed to maintain the peri-implant soft tissues contour.

**Trial registration:**

The current trial was retrospectively registered at Clinical trials.gov (ID: NCT05975515, Date: 27-July-2023).

## Background

Preservation of the labio-palatal ridge dimension is the prime goal following immediate implant placement (IIP) in the anterior maxilla[1]. Nevertheless, it is well established that hard and soft tissue volume are substantially altered at extraction sites particularly in the esthetic zone [2]. Hence, achieving long-term soft and hard tissue stability with IIP has been considered a challenge in everyday clinical practice [3]. The anterior maxilla often has a thin gingival phenotype [4],thus, mid-facial gingival margin changes are considered the most prevalent complication after IIP as reported by previous studies and systematic reviews [5–7].

Various surgical techniques including: proper implant positioning [8], flapless protocol [9], use of bone grafts [10–12], simultaneous connective tissue grafting (CTG) [13–15], socket shield technique [16, 17], and the use of customized healing abutments (CHA) [18, 19], have been proposed over the years as strategies for maintaining the health and stability of the peri-implant tissues in the esthetic zone. The addition of bone grafting material with IIP was developed to decrease the unfavorable labio-palatal ridge collapse following tooth extraction [9]. While simultaneous use of CTG with IIP in the esthetic zone, is presumed to be the gold standard for achieving dimensional soft tissue stability [10–12]. Whereas the utilization of a CHA aimed to reproduce the precise contours of the cervical root area and maintain the soft tissue contours during osseointegration and healing of the peri-implant mucosa [13].

To the best of the authors’ knowledge, this is the first randomized clinical trial comparing the explicit effect of using either bone graft, CTG, or CHA alone following IIP after 1 year. Given the existing gap of knowledge, the aim of this investigation was to evaluate the midfacial gingival margin changes as the primary outcome, besides labial soft tissue volume alterations, radiographic and clinical outcomes. The null hypothesis was that there was no significant difference among the three studied groups in decreasing the midfacial gingival margin changes.

## Methods

### Study population and ethical approval

**Fig. 1:**
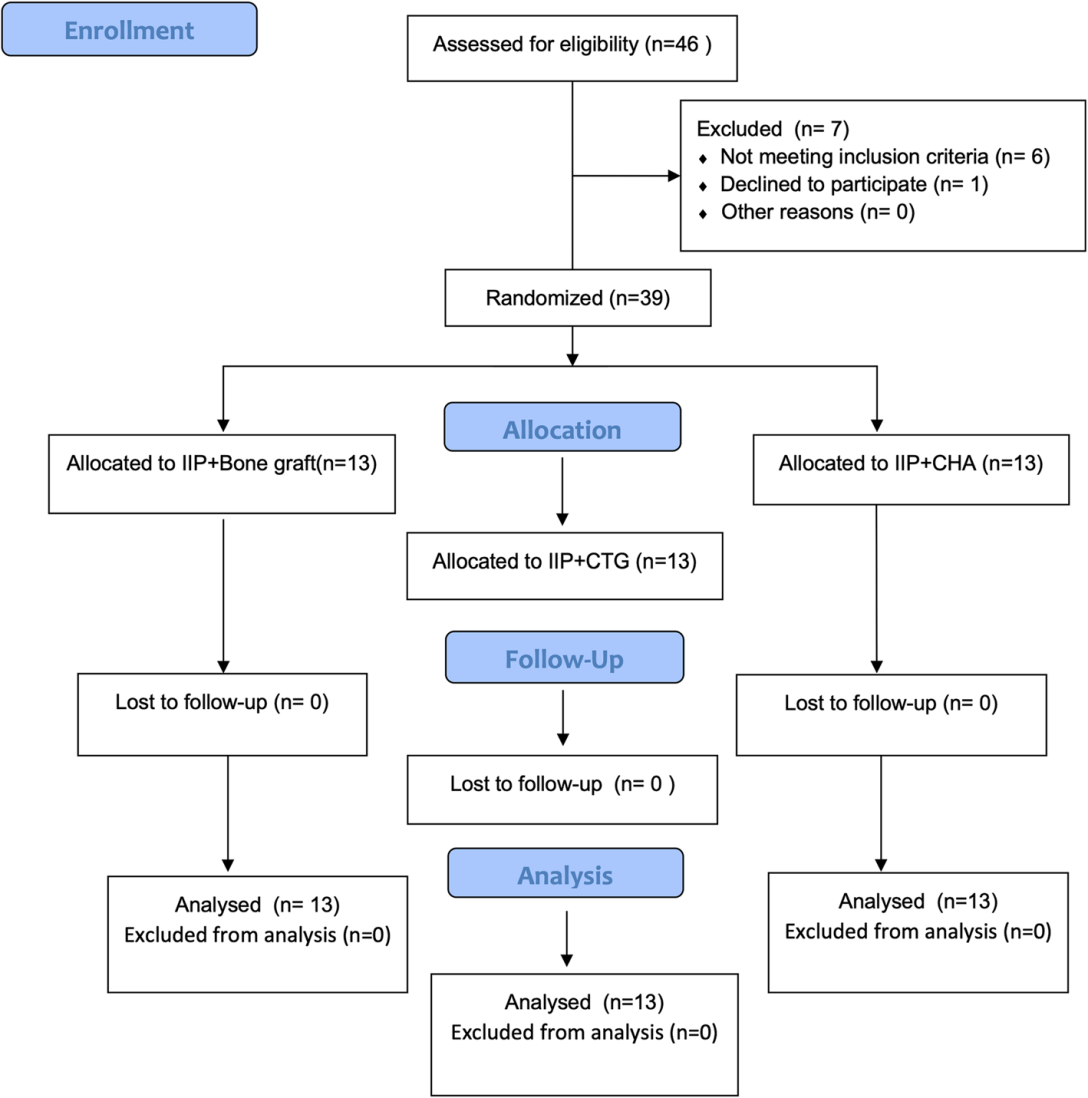
CONSORT flow diagram

This randomized clinical trial was registered at Clinical trials.gov (ID: NCT05975515), and approved by the Research Ethics Committee, Faculty of Dentistry, Cairo University (Approval number 16–1-22). It was conducted according to the Helsinki declaration principles of 1975, as revised in 2013 and reported according to CONSORT guidelines [14] Fig. 1. The trial included 39 non-restorable maxillary teeth indicated for extraction (from 2nd premolar to 2nd premolar) in 39 patients (33 females and 6 males, age range 21–54 years) recruited from the outpatient clinic of Periodontology Department Faculty of Dentistry, Cairo University and from a private practice (International dental clinic) in Cairo, Egypt between February 2022 and April 2022. All the recruited patients were selected according to a predefined inclusion criteria including: (1) Non-restorable single-bounded maxillary tooth in the esthetic zone; (2) Thin labial plate of bone (< 1 mm), assessed by preoperative cone beam computed tomography (CBCT); (3) Intact socket walls (interproximal and labial bone) with sufficient apical bone to ensure implant primary stability with a minimum of 35 N cm insertion torque; (4) Labio-palatal socket dimension measured from the CBCT axial cut at mid-crestal part ≥ 5 mm [15]; and (6) Intact thick gingival tissue with at least 2 mm band of keratinized tissue [4]. Exclusion criteria were as follows: (1) Smokers; (2) Patients with active periodontitis and history of periodontitis; (3) Pregnant females; (4) Systemic diseases that contraindicate implant placement and perio-plastic surgeries; (5) Parafunctional habits such as bruxism or clenching; (6) History of radiotherapy or chemotherapy within the past 2 years; (7) Patients with active infection related to the implant site and (8) Fractured interdental or palatal bone during tooth extraction. Eligible patients agreed to sign a written informed consent to participate in this investigation and were informed about the surgical procedure and trial timeline.

### Sample size calculation

A total sample size of 33 patients was calculated to detect a minimum clinically significant difference of 0.5 mm in mid-facial gingival margin change between CHA with or without bone graft using 80% power, 5% significance level and a standard deviation of 0.4 obtained from previous data [16]. This number was increased to total of 39 patients to compensate for 20% possible losses during the follow up period. The sample size was calculated by PS program (Power and sample size program: biostat.mc.vanderbilt.edu/twiki/bin/view/Main/Power Sample Size) [12, 15, 17].

### Randomization and blinding

Sequence generation was executed using simple randomization by a random allocation software [18] by an investigator (GN) who was not involved in neither recruitment nor treatment procedures. Allocation concealment was conducted by the same investigator (GN) using sequentially-numbered, opaque, sealed envelopes handled to the surgeon (AA) who did not open them until the beginning of the surgical procedure. After recruitment, eligible participants were randomly allocated into three equal parallel groups (*n* = 13) with a 1:1:1 allocation ratio based on the generated sequence to receive either; CHA with bone graft (IIP + Bone graft) (control group), or CHA with CTG (IIP + CTG) (Intervention 1), or CHA only (IIP + CHA) (Intervention 2). The outcome assessor (SN) and data analysts (GN) were blinded, yet due to the nature of the intervention, the participants and the surgeon (AA) could not be blinded.

### Preoperative assessment

Full mouth supra and subgingival debridement was performed on all recruited patients with well-explained oral hygiene instructions. Pre-extraction measurements were recorded from preoperative CBCT records of the maxillary arch (Cranex® SOREDEX, Finland). All data were obtained in a DICOM format and imported to OnDemand3D®App software (Cybermed, Seoul, Korea) [15].

### Surgical procedures

**Fig. 2:**
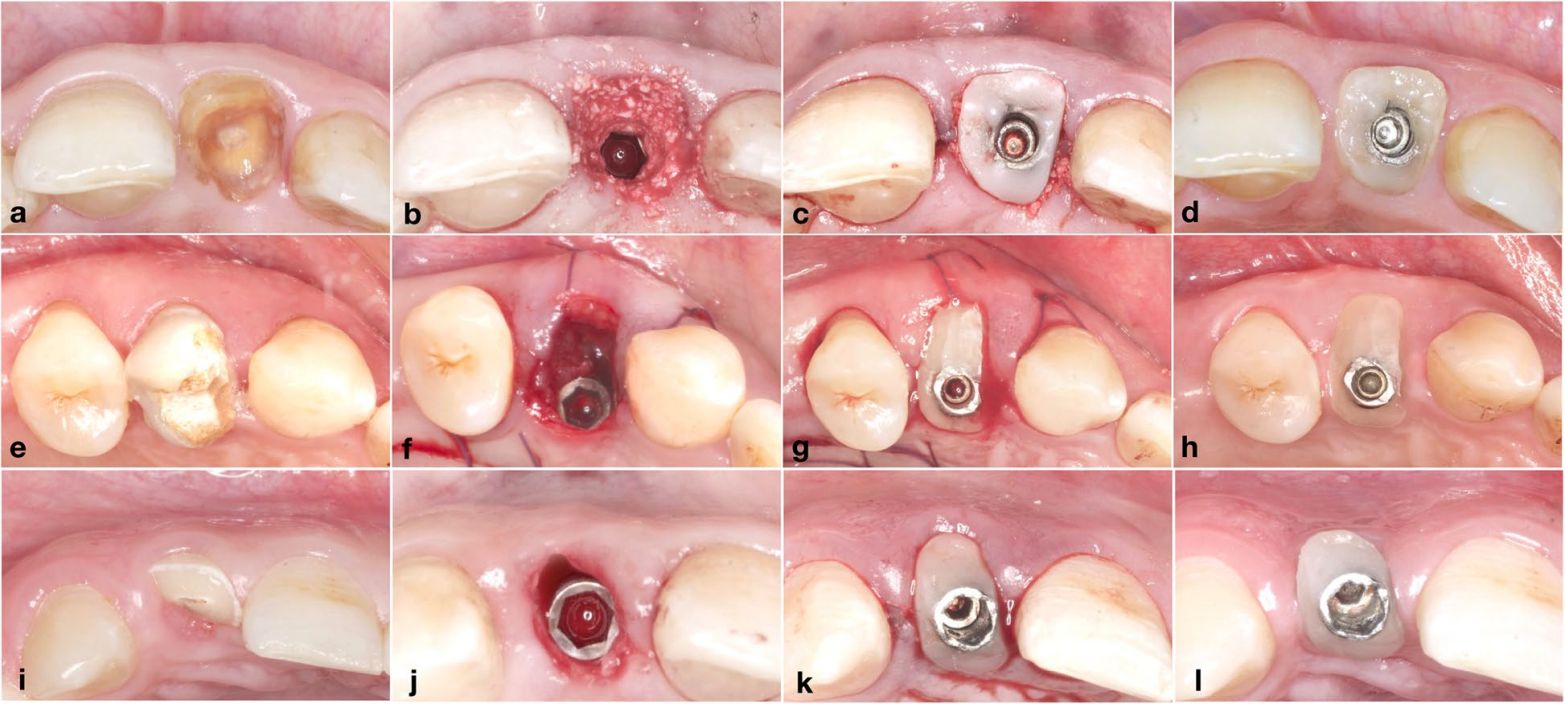
Surgical procedures: IIP + Bone graft group (a–d); (**a**) pre-operative clinical photograph showing occlusal view of upper left central incisor, (**b**) occlusal view showing IIP with bone graft filling the labial gap, (**c**) customized healing abutment screwed to the implant, (**d**) occlusal view showing the labial contour 12 months post-operative. IIP + CTG group (e–h); (**e**) pre-operative clinical photograph showing occlusal view of upper right first premolar, (**f**) occlusal view showing IIP with simultaneous CTG tucked in the labial pouch, (**g**) customized healing abutment screwed to the implant, (**h**) Occlusal view showing the labial contour 12 months post-operative. IIP + CHA group (i-l); (**i**) pre-operative clinical photograph showing occlusal view of upper right lateral incisor, (**j**) occlusal view showing IIP only and the horizontal labio-palatal gap, (**k**) Customized healing abutment screwed to the implant, (**l**) Occlusal view showing the labial contour 12 months post-operative

**Fig. 3:**
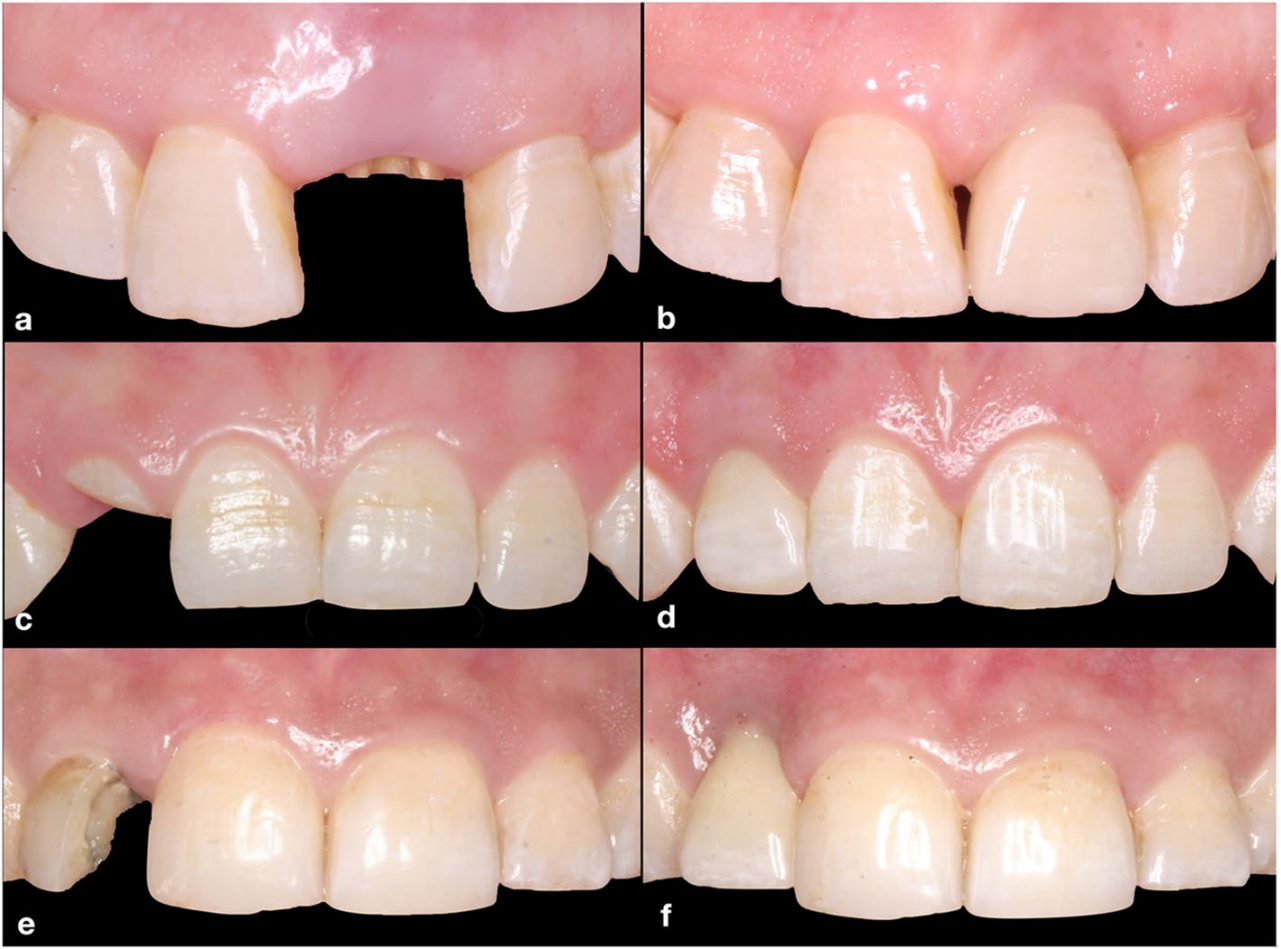
Clinical photographs showing pre- and one year postoperative: IIP + Bone graft group (a-b); (**a**) clinical photograph showing pre-operative labial view of upper left central incisor, (**b**) clinical photograph showing labial view of the implant crown after one year. IIP + CTG group (c-d); (**c**) clinical photograph showing pre-operative labial view of upper right lateral incisor, (**d**) clinical photograph showing labial view of the implant crown after one year. IIP + CHA group (e–f); (**e**) clinical photograph showing the pre-operative labial view of upper right lateral incisor, (**f**) clinical photograph showing labial view of the implant crown after one year

In the current investigation the surgical procedures were executed by an expert surgeon (AA). Description of the surgical procedures and postoperative clinical photographs after 1 year are shown in Figs. 2 and 3 for the three studied groups. Atraumatic extraction of the non-restorable tooth was executed, the integrity of the labial plate was then confirmed. Osteotomy site preparation and implant drilling sequence were performed according to the manufacturer’s instructions. Flapless protocol was implied, where a tapered screw-vent bone level implant (Zimmer implant TSV, Indiana, USA) was inserted into the extraction socket [12]. The implant platform was positioned 3–4 mm apical to the labial gingival margin and 1.5 mm sub-crestal or equicrestal to the labial bone.

Following implant placement, randomization sequence with allocation to either IIP + Bone graft, or IIP + CTG or IIP + CHA group was revealed. Participants assigned to the IIP + Bone graft group received demineralized bovine bone graft (Bio-Oss, Geistlich Pharma AG, Wolhusen, Switzerland). Particulate bone grafts were packed in the gap between the implant and the labial plate of bone, followed by screwing a previously fabricated CHA with the proper torque to the implant [15]. Regarding the IIP + CTG group, patients received pouch CTG [12]. CTG was then stabilized at the desired position in the labial pouch using a resorbable suture material. Subsequent to CTG suturing, a CHA was screwed with proper torque to the implant. Meanwhile, patients assigned to the IIP + CHA group received an immediate implant followed by CHA only. The CHA was fabricated by adding flowable composite to the temporary cylindrical abutment following the shape of the socket at the free gingival margin (FGM) [12].

Antibiotics were prescribed to each participant, including 500 mg metronidazole (Flagyl: Metronidazole 250 mg. Tablets, Alexandria Pharmaceutical CO., Alexandria, Egypt). and 500 mg Amoxicillin (E-mox 500 mg Cap., E.I.P.I.C.O., Egyptian Int. Pharmaceutical Industrial Co., A.R.E.) every 12 h, 24 h preoperatively and after the surgical procedure for 5 days. In addition, non-steroidal anti-inflammatory drugs (Brufen, 400 mg tablets, Kahira pharm. Co. Egypt) was prescribed whenever needed to relief the postoperative pain. and chlorohexidine mouth wash were prescribed to each patient, along with well explained postoperative care and instructions. After 1 year, the CHA was disconnected and an impression was taken using a closed tray technique. The CHA was connected again after the impression to counteract the natural reduction of the mucosal tissues. One week later, a cement retained definitive crown was delivered.

### Clinical measurements

Full‐mouth plaque score (FMPS) [19] and full‐mouth bleeding score (FMBS) [20] were both measured pre-extraction and 1 year post-implant placement. Pain and discomfort were assessed using the visual analog scale (VAS) (1–100) as recorded by the patient 1, 3 and 7 days after the surgery [21]. Implant survival criteria were chosen according to Albrektsson et al. [22].

### Volumetric analysis

The volumetric analysis in this clinical trial was performed via 3D viewer software (Exo cad, Fraunhofer Institute, Germany) [12, 23, 24]. Conventional Polyvinyl Siloxane impressions (Hydrorise Putty and light, additional silicone, Zhermack SpA, Italy) were taken at baseline, 3-, 6- and 12-months post-operative for each patient. The stone casts produced were optically scanned using lab scanner (Shape lab scanner E series, Copenhagen, Denmark). Digital surface models were then created in standard tessellation language (STL) format, all STL files were imported to a digital software for analysis. Throughout the follow-up period, the best-fit algorithm was used to superimpose digital surface models when comparing each area of interest (AOI) [12].

**Fig. 4:**
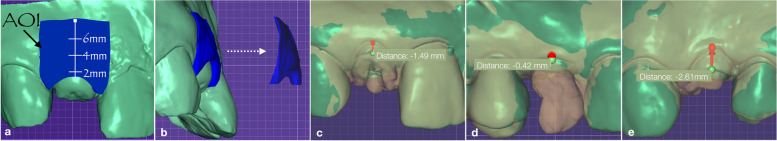
Volumetric Analysis by superimposition of the scanned casts with best fit algorithm; (**a**) frontal view of the scanned cast at 12 months showing the limits of the AOI included in the linear subtraction and volumetric analysis procedure measuring the labial soft tissue contour changes at 2, 4, and 6 mm from FGM, (**b**) profile view of the scanned cast showing surface volume analysis procedure used in evaluating total volume change after 12 months postoperative (blue area), (**c**) frontal view of scanned casts showing linear measurement of mid-facial gingival marginal changes (-1.49 mm) in the IIP + Bone graft group at 12 months postoperative, (**d**) frontal view of scanned casts showing linear measurement of mid-facial gingival marginal changes (-0.42 mm) in the IIP + CTG group at 12 months postoperative, (**e**) frontal view of scanned casts showing linear measurement of mid-facial gingival marginal changes (-2.61 mm) in the IIP + CHA group at 12 months postoperative

The trial outcomes were measured as follows Fig. 4: Mid-facial gingival margin changes from scanned casts pre- and post-operatively as the primary outcome. It was recorded by measuring the distance between the level of soft tissue margin mid-facially postoperatively and a reference line which connected the labial soft tissue margins of the adjacent teeth preoperatively, at 3, 6 and 12 months. Linear changes in labial soft tissue contour at levels of 2 mm, 4 mm, and 6 mm from FGM, was calculated by a linear subtraction method (mm) from superimposition of the baseline and the scanned casts at 3, 6 and 12 months postoperative. Mesial and distal interdental tissue (IDT) height changes (mm) were also measured using the same linear subtraction method at 3, 6 and 12 months postoperative. Total volume change (mm3) was calculated at 12 months postoperative via surface volume analysis within the AOI for each patient.

### CBCT measurements

Postoperative CBCT evaluation was performed on all patients after 1 year. The amount of bone labial to the implant was measured at three levels below the labial bone crest; 0 mm, 2 mm and 5 mm, crestal bone level changes were recorded as well [15]. To guarantee standardization and duplicability of the CBCT images during comparisons, superimposition of DICOM file sets of each patient were done using Fusion module software (OnDemand3D ver, 1.0.9, Cybermed), which allowed sub-voxel accuracy [15, 25].

### Statistical & Power analysis

Numerical data were explored for normality by checking the distribution of data and using tests of normality (Kolmogorov–Smirnov and Shapiro–Wilk tests). Data were presented as mean, standard deviation (SD), median and range values. For parametric data, one-way analysis of variance (ANOVA) test was used to compare between the three groups. Repeated measures ANOVA test was used to compare between the three groups as well as to study the changes by time within each group. Bonferroni’s post-hoc test was used for pair-wise comparisons when ANOVA test was significant. For non-parametric data, Kruskal–Wallis test was used to compare between the three groups. Friedman’s test and Wilcoxon-signed rank test were used to study the changes by time within each group. Dunn’s test was used for pair-wise comparisons when Friedman’s test was significant. Qualitative data were presented as frequencies and percentages. Chi-square and Fisher’s Exact tests were used to compare the three groups regarding qualitative variables. Spearman’s correlation coefficient was used to determine the correlation between total volume loss and mid-facial gingival margin changes after one year. The significance level was set at P ≤ 0.05. Statistical analysis was performed with IBM SPSS Statistics for Windows, Version 23.0. Armonk, NY: IBM Corp.

## Results

### Clinical measurements

Intra-examiner repeatability for CBCT measurements revealed satisfactory agreement, with an intraclass correlation coefficient value of 0.88 (mean error 0.16 mm). Data for baseline characteristics are shown in Table 1. All recruited subjects completed the 1-year follow up period with no implants lost. No signs of surgical or prosthetic complications were noticed throughout the experimental period. Intergroup differences showed no significant differences in the FMPS and FMBS after 1‐year compared to baseline values in the three groups. Patients treated in the IIP + Bone graft group displayed a significant difference (P < 0.001) between mean ± SD FMPS (%) at baseline (16.38 ± 1.61) and after one year (11.69 ± 1.93); While FMBS (%) showed 9.62 (± 1.89) and 7.54 (± 1.98); with a significant difference between baseline and 1-year (*P* < 0.001). Regarding IIP + CTG group, baseline FMPS (%) showed 18.38(± 2.63) and after one year it was 12.54(± 2.54). While FMBS (%) revealed 9.85(± 2.23) and 7.38 (± 2.36) at baseline and one-year postoperative respectively with a significantt difference between the two time points (*P* < 0.001). Meanwhile, patients treated with IIP + CHA showed FMPS (%) of 17.46 (± 2.50) at baseline with a statistically significantt decrease reporting 11.08(± 2.18) after one year (*P* < 0.001). Whereas FMBS (%) demonstrated a mean(± SD) of 10.15 (± 2.30) at baseline and of 5.92 (± 1.89) one year post-operatively, revealing a significant decrease between the two follow up periods (*P* < 0.001). Regarding pain and discomfort, Patients treated in the IIP + Bone graft group demonstrated pain scores (VAS) with median (range) of 60 (45, 75), 40 (25, 50), and 5 (0, 10) at 24 h, 3 days and 7 days postoperative respectively. Median (range) pain scores (VAS) reported in the IIP + CTG group were 60 (50, 70), 40 (30, 50), and 5 (0, 10) at 24 h, 3 days and 7 days post-operative respectively. IIP + CHA group showed median (range) pain scores of 60 (45, 75), 40 (25, 50), and 5 (0, 10) at 24 h, 3 days and 7 days postoperative respectively. Pain and discomfort, reached their peak 24 h after implant placement and then slowly decreased 3 and 7 days postoperatively, with no significant difference between the three groups.

**Table 1:** Baseline demographic, clinical and radiographic data of the participants included in all studied groups

	**IIP + Bone graft** ***n* = 13**	**IIP + CTG** ***n* = 13**	**IIP + CHA** ***n* = 13**	***P*-value**
Age (years) mean (± SD)	36.4 (± 7.6)	39.2 (± 9.2)	34.7 (± 8.1)	0.39
Male *n* (%)	2 (15.4%)	1 (7.7%)	3 (23.1%)	0.85
Female *n* (%)	11 (84.6%)	12 (92.3%)	10 (76.9%)	
Implant length (mm)				
11.5 (*n*)	4	6	5	-
13 (*n*)	9	7	8	-
Implant diameter (mm)				
3.7 (*n*)	4	8	6	-
4.1 (*n*)	9	5	7	-
Number of surgeries by site				
Central incisor (*n*)	1	2	-	-
Lateral incisor (*n*)	2	2	1	-
Canine (*n*)	1	-	2	-
First premolar (*n*)	7	7	8	-
Second premolar (*n*)	2	2	2	-
Labio-palatal socket dimension mean ± SD	5.78^a^ ± 1.10	5.71^a^ ± 1.12	6.39^a^ ± 1.19	0.26
Initial labial bone plate thickness mean ± SD (mm below the buccal bone crest)				
0	0.85^a^ ± 0.06	0.85^a^ ± 0.06	0.86^a^ ± 0.08	0.81
2	0.82^a^ ± 0.09	0.81^a^ ± 0.09	0.83^a^ ± 0.01	0.91
5	0.76^a^ ± 0.15	0.79^a^ ± 0.16	0.74^a^ ± 0.14	0.77
Insertion torque N/cm mean (± SD)	45.00^a^ ± 5.00	43.46^a^ ± 6.25	45.76^a^ ± 4.00	0.51
Implant survival *n* (%)	13 (100)	13 (100)	13 (100)	1.00

*SD* Standard deviation

Means have different letters are statistically significant

### Volumetric analysis

Table 2 shows linear and volumetric changes recorded for all groups throughout the study including; mid-facial gingival margin changes (mm), labial soft tissue contour changes (mm), interdental tissue height changes (mm) and total volume change (mm3).

**Table 2:** Mean ± SD of mid-facial gingival margin, linear labial soft tissue contour, IDT height and total volume changes in all studied groups throughout the experimental period

	**IIP + Bone graft *n* = 13**	**IIP + CTG *n* = 13**	**IIP + CHA *n* = 13**	***P*-value**
Mid-facial gingival margin change (mm)				
0–3 months	-0.83^a^ ± 0.32	-0.72^a^ ± 0.44	-1.20^b^ ± 0.46	0.02*
0–6 months	-0.83^a^ ± 0.34	-0.73^a^ ± 0.35	-1.30^b^ ± 0.28	0.03*
0–12 months	-0.98^a^ ± 0.37	-0.74^b^ ± 0.48	-1.54^c^ ± 0.70	0.005*
*P*-value	**0.07**	**0.92**	**0.06**	**N/A**
Volumetric change of labial soft tissue 0–3 months (mm below free gingival margin)				
2	-0.55^a^ ± 0.23	-0.24^b^ ± 0.42	-0.87^c^ ± 0.51	0.009*
4	-0.37^a^ ± 0.14	0.19^b^ ± 0.53	-0.55^c^ ± 0.30	0.001*
6	-0.47^a^ ± 0.33	0.43^b^ ± 0.64	-0.47^a^ ± 0.19	< 0.001*
Volumetric change of labial soft tissue 0–6 months (mm below free gingival margin)				
2	-0.67^a^ ± 0.33	-0.21^a^ ± 0.30	-0.94^a^ ± 0.54	**0.53**
4	-0.48^a^ ± 0.21	0.12^a^ ± 0.36	-0.68^a^ ± 0.38	**0.67**
6	-0.56^a^ ± 0.38	0.24^b^ ± 0.56	-0.61^a^ ± 0.26	0.03*
Volumetric change of labial soft tissue 0–12 months (mm below free gingival margin)				
2	-0.84^a^ ± 0.35	-0.13^b^ ± 0.29	-1.07^c^ ± 0.60	0.001*
4	-0.61^a^ ± 0.25	0.12^b^ ± 0.34	-0.88^c^ ± 0.48	< 0.001*
6	-0.78^a^ ± 0.39	0.10^b^ ± 0.57	-0.80^c^ ± 0.35	< 0.001*
Mesial interdental papilla height change (mm)				
0–3 months	-0.58^a^ ± 0.60	-0.70^a^ ± 0.28	-0.76^a^ ± 0.23	**0.53**
0–6 months	-0.73^a^ ± 0.64	-0.66^a^ ± 0.25	-0.87^a^ ± 0.35	**0.32**
0–12 months	-0.94^b^ ± 0.63	-0.71^a^ ± 0.26	-0.92^a^ ± 0.41	**0.12**
*P*-value	0.004*	**0.14**	**0.34**	
Distal interdental papilla height change (mm)				
0–3 months	-0.73^a^ ± 0.25	-0.77^a^ ± 0.18	-0.70^a^ ± 0.19	**0.67**
0–6 months	-0.87^a^ ± 0.37	-0.69^a^ ± 0.31	-0.82^a^ ± 0.26	**0.26**
0–12 months	-1.09^c^ ± 0.44	-0.63^b^ ± 0.45	-0.86^a^ ± 0.36	0.03*
*P*-value	0.009*	**0.16**	**0.26**	
Total Volume change after 1 year (mm^3^)	-42.75^**a**^** ± **13.96	-8.92^**b**^** ± **12.79	-45.51^**a**^** ± **18.79	< 0.001*

Means have different letters are statistically significant

*SD* Standard deviation

^*^Corresponds to statistically significant difference at *P* ≤ 0.05

### Mid-facial gingival margin changes (mm)

Remarkably, IIP + CTG group presented the least change in the mid-facial gingival margin when compared to the other two groups throughout the study period. Although the mid-facial gingival margin changes at 0–3 months and at 0–6 months were not significant when IIP + CTG was compared to IIP + Bone graft, yet, a significant difference was observed when both IIP + Bone graft and IIP + CTG were compared to IIP + CHA (*P* < 0.05). Interestingly, at 0–12 months there was a significant difference regarding the change in mid-facial gingival margin between the three groups (*P* = 0.005). Sites treated with IIP + Bone graft revealed significantly more gingival margin loss (-0.98) compared to IIP + CTG (-0.74) while those treated with IIP + CHA revealed significantly more midfacial gingival margin alterations (-1.54 mm).

### Linear changes in labial soft tissue contour (mm)

Interestingly, sites treated with IIP + CTG showed a statistically significant gain in the mean labial soft tissue contour change after 1 year compared to IIP + Bone graft and IIP + CHA groups throughout the experimental period and at all measured points. Intergroup analysis revealed a significant difference between the three treatment modalities in the linear change from 0–3 months of the labial soft tissue at 2, 4 and 6mm below the FGM (*P* < 0.01). While the labial soft tissue changes from 0–6 months showed no significant difference between the three groups at 2mm and 4mm from the FGM (*P* > 0.05). Nevertheless, at 6 mm from the FGM, the labial soft tissue changes from 0–6 months revealed a statistical significance between IIP + CTG and the other groups (*P* = 0.03), with no significant difference observed between IIP + Bone graft and IIP + CHA. The presented data also showed a statistically significant difference between the three modalities in the labial soft tissue change from 0–12 months at 2, 4 and 6 mm below the FGM (*P* ≤ 0.001) where the IIP + CTG group displayed a significant gain in the labial contour.

### Interdental tissue height change (mm)

The IIP + Bone graft group showed a significant loss in mesial and distal IDT (mm) at 0–12 months compared to 0–3 and 0–6 months (*P* < 0.01), yet no significant difference was observed between the changes at 0–3 and 0–6 months (*P* > 0.05). However, the IIP + CTG and IIP + CHA groups displayed no significant differences throughout the different follow up periods (*P* > 0.05). The current intergroup comparisons showed no statistically significant difference in the mesial IDT height change (mm) between the three treatment modalities throughout the study period (*P* > 0.05). Likewise, the distal IDT height change (mm) showed no significant difference between the three groups at 0–3 months and at 0–6 months (*P* > 0.05). Interestingly, there was a significant difference in distal IDTheight change (mm) at 0–12 months among all the studied groups (*P* = 0.03), with the IIP + CTG group revealing the least change and the IIP + Bone graft group showing the more loss.

### Total volume change (mm3)

The currently presented data demonstrated a significant difference between the three studied groups regarding mean total volume change (mm3) one year post operative (*P* value < 0.001). The IIP + Bone graft group revealed a mean ± SD total volume change of -42.75 ± 13.96 mm3, while IIP + CTG showed a mean ± SD of -8.92 (± 12.79) mm3 whereas a mean ± SD of -45.51 ± 18.79 mm3 was demonstrated in the IIP + CHA group. Remarkably, the IIP + CTG group demonstrated the least total volume loss (mm3) after 1 year compared to the other two treatment modalities.

### Correlation between total volume change (mm3) and mid-facial gingival margin changes

Results of Spearman’s correlation coefficients revealed that in the IIP + Bone graft and IIP + CHA groups there was a statistically significant direct (positive) correlation between the total volume change (mm3) after 1 year and the mid-facial gingival margin changes 0–12 months (*r* = 0.65, *P* = 0.02 and 0.75, *P* = 0.003 respectively). However, there was no statistically significant correlation observed in the IIP + CTG group (*r* =—0.15, *P* = 0.63).

### CBCT measurements

Table 3 shows the recorded CBCT measurements after 1 year.

**Table 3:** Mean ± SD of the bone labial to the implant at different measured levels and crestal bone level changes for all studied groups after one year

	**IIP + Bone graft *n* = 13**	**IIP + CTG *n* = 13**	**IIP + CHA *n* = 13**	***P*-value**
Amount of bone labial to the implant (mm)(mm below the labial bone crest)				
0	3.16^a^ ± 1.04	3.20^a^ ± 1.45	2.55^a^ ± 1.21	**0.34**
2	3.01^a^ ± 1.15	2.89^a^ ± 1.49	2.26^a^ ± 1.24	**0.29**
5	2.96^a^ ± 1.34	2.51^a^ ± 1.53	1.91^a^ ± 1.33	**0.17**
Overall amount of bone labial to the implant (mm)	3.06^a^ ± 1.14	2.87^a^ ± 1.47	2.24^a^ ± 1.24	**0.26**
Crestal bone level changes mm (vertical changes)	0.24^a^ ± 1.12	-1.17^b^ ± 0.64	-1.92^b^ ± 1.05	< 0.001*

Means have different letters are statistically significant

*SD* Standard deviation

^*^Corresponds to statistically significant difference at *P* ≤ 0.05

### Amount of bone labial to the implant

The mm amount of bone labial to the implant was assessed at three points, 0, 2 and 5 mm apical to the labial bone crest. The present results demonstrated that bone filled the gap in the three studied groups without a significant difference between them (*P* = 0.26). The mean ± SD mm overall amount of bone formed labial to the implant was 3.06 ± 1.14, 2.87 ± 1.47 and 2.44 ± 1.24 in the IIP + Bone graft, IIP + CTG and IIP + CHA respectively.

### Crestal bone level changes

The mean ± SD crestal bone level changes after 1 year in the IIP + Bone graft, IIP + CTG and IIP + CHA groups were 0.24 ± 1.12, -1.17 ± 0.64 and -1.92 ± 1.05 mm respectively with a significant difference observed between the three groups (*P* < 0.001). Remarkably, the IIP + Bone graft group showed a significant gain in crestal bone level changes compared to both IIP + CTG and IIP + CHA. Although sites treated with IIP + CTG revealed less crestal changes after 1 year compared to those treated with IIP + CHA, yet, this difference was not statistically significant (*P* > 0.05).

## Discussion

This randomized clinical trial aimed to assess the peri-implant profilometric, radiographic and clinical outcomes one year following flapless IIP with three different treatment modalities either bone graft till labial bone crest, CTG tucked labial to the extraction socket or CHA solely at sites with thin labial plate of bone (< 1mm) in the esthetic zone. Results of this clinical trial showed that sites treated with IIP + CTG group had the least significant midfacial gingival margin change after 1 year (-0.74mm) compared to those treated with IIP + Bone graft (-0.98mm) and IIP + CHA, which revealed the most alterations (-1.54mm).

The currently presented findings were comparable to Yuenyongorarn [26], who reported a significantly less midfacial gingival level change after 12 months after using bone grafts with IIP (0.77 mm) compared to non-grafted group (1.35mm). The authors proposed that placement of bone graft into the implant-socket gap dimension may lessen, but not prevent, the midfacial gingival margin changes. Likewise, Bittner et al. [27] presented less buccal soft tissue vertical height in bone graft group compared to non-grafted sites, yet the difference was insignificant. The present results were also in line with a recent clinical trial observing a mid-facial gingival margin change of -0.6 mm one year after using bone graft with IIP [23]. Although van Nimwegen [28] reported superior -0.48mm mid-facial mucosal levels in the grafted sites, this might be attributed to the fact that measurements were taken after definitive crown placement which might affect the gingival level. Numerous clinical trials and systematic reviews [11, 12, 23, 29–31] suggested that IIP + CTG could significantly stabilize the midfacial peri-implant mucosal level (< 1mm loss) compared to IIP without CTG which support the present observations. Furthermore, the currently reported mean midfacial gingival margin changes in the IIP + CTG group (-0.74mm) were in accordance with Fettouh et al. [12], where the authors showed a mean change of (-0.86mm) in their CTG group. While Levine et al. [32] reported no significant difference between CTG and non-grafted sites regarding the amount of midfacial mucosal remodeling at the implant sites, yet the frequency of sites with mid-facial mucosal remodeling ≥ 0.5 mm was higher in sites without CTG. Nevertheless, other trials revealed limited advantages of using CTG with IIP compared to other graft materials [33] or no graft [34]. There is a current controversy in the literature concerning the benefits of using CHA alone in maintaining the peri-implant soft tissue. Fernandes et al. [35] showed -0.55mm after using CHA solely with flapless maxillary IIP and explained their findings owing to the pressure and absence of space caused by CHA at the facial mucosa. In contrast, Lenz et al. [36] in their recent systematic review concluded that CHA might maintain the emergence profile at immediate implant sites with no significant loss of soft and hard tissue. These inconsistent findings might be due to the different surgical protocols and different measurement methodologies used to assess the mid-facial gingival margin alterations.

Although IIP in the aesthetic zone have been widely discussed in the literature, yet there are scarce data reporting the linear and volumetric soft tissue changes [12, 37]. In the current trial digitalized soft tissue analysis provided the detailed net linear and volume alterations to evaluate the esthetic outcomes. Labial soft tissue contour changes (mm) showed a significant difference between the three studies modalities after one year, where the IIP + CTG sites showed the least labial soft tissue contour reduction compared to the other treatment protocols. Consistent findings were observed in previous trials reporting conserved labial soft tissue contour and gingival profile after using IIP + CTG compared to IIP without CTG [26, 38, 39]. Accordingly, it might be speculated that CTG aided in limiting the post-extraction soft tissue alterations, preserving the labial soft tissue contour. The findings presented herein demonstrated a significant difference in the total volume change (mm3) among the three studied groups after 12 months. Remarkably, sites treated with IIP + CTG showed the least total volume loss (-8.92 ± 12.79) mm3 while IIP + CHA group revealed the highest volume loss (-45.51 ± 18.7) mm3, followed by IIP + Bone graft group (-42.75 ± 13.96 mm3). Similarly, Fernandes et al.[23] and van Nimwegen et al. [28] reported less total volume loss (-8.4 and -9.32 mm3 respectively) at sites treated with IIP + CTG. Despite that Guglielmi et al. [39] reported noticeable soft tissue contour enhancement in the IIP + CTG sites (6.76 mm3) after 6 months, this discrepancy might be explained by the shorter follow up period, as well as using flap elevation approach in their study. Recently, Fettouh et al. [12] observed a mucosal total volume loss of 36.04 mm3 after using bone graft compared to 15.6 mm3 loss using CTG with IIP.

There were notable clinical alterations in both mesial and distal IDT among the three groups after one year. Interestingly, the IIP + CTG group demonstrated the least IDT height changes (-0.71 ± 0.26 mesially and -0.63 ± 0.45 distally) compared to the other groups. This was in accordance with Fernandes et al.[23], who reported that IIP + CTG sites had approximately three times less IDT height variations when compared to bone-grafted sites, yet, with no statistical significance. Likewise, Valles et al.[40] did not find a significant difference in IDT height variations between grafted and nongrafted IIP sites, still the IDT height was nearly preserved at sites treated with IIP + CTG throughout the follow-up period. It might be suggested that the presence of a thick peri-implant mucosa resulted in a substantially more favorable interdental tissue volume/fill.

There is well-established evidence that the presence of a sufficient labial bone plate (> 1mm) is fundamental for long-term implant survival rates, functional and esthetic success [23, 41]. In accordance, the present trial observed that sites treated with bone graft, CTG and CHA showed an acceptable mean amount of bone formed labial to the implant (3.06 ± 1.14, 2.87 ± 1.47 and 2.24 ± 1.24mm respectively) with no significant difference between them. These results were in line with Fettouh et al. [15] who reported 2.7 and 2.5 mm mean amount of bone labial to the implant in grafted and non-grafted sites respectively after one year. Meanwhile, Levine et al. [32] who included only non-restorable central incisors, showed inferior mean amount of bone labial to the implant (1.5 mm) after using CTG with IIP. In accordance with the abovementioned clinical trials and the present findings, the newly formed bone labial to the implant is dependent on the initially formed and maintained blood clot rather than the use of a regenerative material [15]. Moreover, the present observations revealed a significant mean ± SD mm crestal bone gain (0.24 ± 1.12) at sites treated with IIP + Bone graft, compared to IIP + CTG and IIP + CHA groups which showed crestal bone loss of -1.17 ± 0.64 and -1.92 ± 1.05 respectively. This was in line with the 6th EAO Consensus which reported that “the present scientific evidence does not consistently demonstrate a benefit of soft tissue augmentation procedures in terms of marginal bone level changes” [42]. In contrast, Guglielmi et al. [39] showed 0.6 mm mean marginal bone loss after using CTG with IIP, similarly Zuiderveld et al. [43] showed less marginal bone loss (0.1mm) in the CTG group. This inconsistency might be attributed to the use standardized digital intraoral radiographs for measurements in the later study.

The current statistical analysis revealed a positive correlation between the total volume change (mm3) and the mid-facial gingival margin changes (mm) in both the IIP + Bone graft and IIP + CHA groups, however, the IIP + CTG group showed no significant correlation. Interestingly, the IIP + CTG group displayed the least midfacial gingival margin change (mm) and total volume change (mm3) after 1-year compared to the other treatment modalities. Whereas the IIP + Bone graft and IIP + CHA groups exhibited almost five folds more total volume loss (mm3) than the IIP + CTG group. Hence, this clinical trial might suggest that having thick soft tissue at baseline in the three groups does not guarantee having an acceptable final supra-crestal tissue volume. The correlation observed herein might assume a direct relationship between the total volume change and mid-facial gingival margin alterations at the “supra-implant complex” area. In a more clinical sense, as the peri-implant soft tissue volume decreases, the mid-facial gingival margin level might also recede in an apical direction resulting in a peri-implant midfacial recession.

To the best of the authors' knowledge, this is the first randomized clinical trial assessing three different treatment modalities with IIP, integrating the linear subtraction analysis of the midfacial gingival margin (mm) and labial soft tissue (mm) changes, along with the total volumetric alterations (mm3) and hard tissue evaluation for better understanding of the “supra-implant complex” tissue behavior [12]. The statistical results of this clinical trial reject the null hypothesis of having no significant difference between IIP + Bone graft, IIP + CTG and IIP + CHA regarding mid-facial gingival margin changes after 1-year.

Based on the above-mentioned findings, the clinical implementation of the current data highlights the importance of the “supra-implant complex” as an influential factor for maintaining the integrity of the peri-implant soft tissue. Further trials are warranted to consider the peri-implant soft tissue volume change as an esthetic parameter at IIP sites in the esthetic zone. Future studies matching the DICOM data with STL files are recommended to analyze the hard and soft tissue interactions. Moreover, clinical studies correlating the implant position and its impact on the supra-implant complex are recommended.

## Conclusions

Within the limitations of present trial, the use of CTG along with IIP in the esthetic zone might reduce the mid-facial gingival margin alterations (mm) by decreasing the overall total volume loss (mm^3^) in the “supra-implant complex” area. Furthermore, using CHA alone with IIP failed to act as a physical support to the peri-implant soft tissue. This might be attributed to the absence of a biological attachment between the CHA and the surrounding tissues. Despite that there was no significant difference in the mean amount of bone labial to the implant among the three studied groups, total volume change revealed a clinically significant difference with the CTG group presenting the least total volume loss. This might denote that the soft tissue volume at the “supra-implant complex” area is totally independent of the bone labial to the implant.

## Data Availability

The data that support the findings of this study are available from the corresponding author upon reasonable request.

## Abbreviations

IIP: Immediate implant placement
CTG: Connective tissue graft
CHA: Customized healing abutment
CBCT: Cone beam computed tomography
3D: Three-dimensional
FMPS: Full‐mouth plaque score
FMBS: Full‐mouth bleeding score
VAS: Visual analog scale
STL: Standard tessellation language
AOI: Area of interest
FGM: Free gingival margin
IDT: Interdental tissue
ANOVA: Analysis of variance
